# Lectures on the Processes of Repair and Reproduction after Injuries

**Published:** 1849-10

**Authors:** James Paget

**Affiliations:** Professor of Anatomy and Surgery to the College


### ANATOMY AND PHYSIOLOGY.

*Lectures on the processes of Repair and Reproduction after Injuries. Delivered at the Royal College of Surgeons of England.* By JAMES PAGET, F. R. C. S., Professor of Anatomy and Surgery to the College.

### LECTURE I.

*Mr. President and Gentlemen,*—There are many considerations which would have led me to choose that which has been announced as the subject of these lectures; but the chief of them was, that the preparations in our Museum which illustrate these processes, are next in order to those that suggested the subjects of two former courses ; while another reason was, the advantages which might result from such a course, as supplemental to those of my colleague, Professor Owen. My mind would gladly stop for a moment in contemplation of those perfect works of nature of which he has discoursed, and with reluctance turn from them to the consideration of the losses to which they are subject, and to processes of repair which they undergo. Yet, in considering the power here exerted, we shall find that it is the same which is seen in bringing to perfection the impregnated germ. For we cannot form a just conception of the nature of these processes, unless we admit that in each perfect organism some measure of that power is exerted which operated to produce the germ. I shall, therefore, ask your permission to occupy the time of this first lecture chiefly with the consideration of this principle. It is not a new doctrine, for the theory of *nisus formativus* of Blumenbach, and that of Müller, might be expressed again in nearly the same words as I have used ; and yet some excuse seems to be necessary for arguing now that such a doctrine should be received. For the general tone of modern physiology is opposed to it, and of late years, especially, the cell-theory of Schleiden and Schwann, and the seeming accuracy which the progress of organic chemistry has given to our notions concerning assimilation, appear to have satisfied most physiologists that we have, in these, sufficiently good hypotheses for the explanation of the maintenance of the body, by nutrition. If it were so, we might need no other hypothesis for the explanation of repair and reproduction ; but I think, that both in these, and in the processes of common nutrition, a power is in exercise which is not admitted by the cell-theory, or by organic chemistry—a power continuous with that manifested in the germ, and acting in all essential things like it.

The characteristic property of an impregnated germ is, that when placed in favourable circumstances, all the materials of which it first consists, and all that it appropriates, are developed according to the same method as was observed in the developement of its progenitors. Among all the marvels of the laws of developement that my colleague has detailed, none, I think, appeared more marvellous than the seeming tenacity of purpose with which each germ passes on to the perfection of its specific form. However vast its power of multiplication and increase—however various its metamorphoses—however near some may approach the perfect characters of another species—or, however much alike all germs may be in their first stage,—yet, through all these things, each germ, self-impelled, passes on to the formation of a being like the parent.

The constancy of this result justifies the expresion, that every impregnated germ has, in itself, the power to develope itself into the perfection of an appropriate specific form from the materials of which it consists, and this we may call the "power of the germ," or "germ-power."

Now, there are certain characters marking the mode in which the germ developes itself ; and by these we may determine the difference between the operations of the germ-power and that of any other process in the living being.

The first characteristic is the limits it has to observe. As surely as the germ tends always to the attainment of perfection, so it is strictly limited to certain rules of time, and space, and mode of progress. The germ rarely falls short of its designed form, but it never goes beyond it,—no creature is known to attain by developement a higher specific form than its parents had ; and the differences of size, to which by growth the different individuals of a species may attain, are circumscribed in narrow limits. So, too, are the length of life, and, in narrower limits still, the periods and methods of developement. Perfection, whether in the whole body, or in any of its parts or tissues, seems to be attainable only through certain forms appropriate to each species ; and from which, if it deviates, it never attains that specific form. then, wherever, this obtains, we may suppose a power is in operation like that of the germ power.

Secondly, it seems to be characteristic of the power of the impregnated germ, that it has the capability of subordinating to one design a number of concurrent but different processes. It is only in few and trivial instances that we can regard the developement of one part as the consequence of the developement of another. They are independent agencies. Thus, the primordial heart and blood-vessels of the embryo together, proceed in regular progress, but quite independent. There is also a concurrence in the developement of each of the several parts of a limb ; yet each limb, and each part is developed independently. And, again, when a new roof of blood-vessels is to be effected, it is manifest that there must be concurrent developement of both, in order that they may exactly meet and coalesce ; but the developement of the one is in no sense a consequence of the developement of the other. The process of combining material thus is so characteristic of the power of the germ, that wherever it is manifested, we may use it as a test of the germ power.

The third characteristic is, that it is developed in all its parts without the presence of any model. And here the power of the germ is different from that seen in the processes of assimilation ; for here all the materials are formed after the pattern of the part in which it is inserted ; it is supposed, that the plasma around it or near it takes the same form. Now, in the germ, the several parts may exist ; but, none of them as a rudiment or model, and no single part is formed in precise imitation to the one already present. If, then, in subsequent life, we find instances in which parts are developed into definite forms, but still distinct parts, we may assume that it is so by the same power that was exerted on the impregnated germ. Here we come to the common doctrine of the maintenance of the body by nutrition. The physiological doctrine seems to be, that each structure in the body has the power of taking from the blood certain appropriate materials, and of assimilating themselves to it ; or else, that it produces cytoblasts or germinal centres. Thus :—1st. That each part of the body has the power of abstracting materials from the blood, of assimilating themselves to it, and incorporating themselves into its structure—the power of giving life to that part which is amorphous. 2d. That each part gives off that which may serve for the developement of another part like it, and which may fill its office

Doubtless, both these may be proved to be in operation; for example, we may prove assimilation by this,—that any change which has passed from the blood may be maintained in that blood, although all the parts may be renovated. Thus, the poison of small-pox being inserted into the blood, poisons the whole of it; and yet the blood is not as it was before; for now it is no longer amenable to that which before disturbed it. Here we have a proof of the assimilative force in the blood; for there is no other mode of explaining this fact, which shows that a part attacked by disease is no longer what it was before, except by admitting that the blood has the sole power of assimilating all new particles which are formed after such disease; all the blood is formed according to the altered model. And so we explain the force exerted in the succession of developements of tissue-germs, as in the case of the teeth. As the first tooth is developed, we recognize in the capsule all the material necessary for the developement of the second; and so of the hair,—each hair pulp and capsule gives off another germ which serves for the developement of its successor; and it is clear that this same power is exerted in the living body;—so there can be no question that in the living body there is constantly exercised a power of assimilation, so that the new material may take the characteristics of that into which it is to be inserted.

Still I think such forces are not sufficient for the maintenance of the body in its perfection; and that, even where they do tend towards that end, they act in subordination to that power which we recognize as determining the impregnated germ in its developement. For, in the first place, there are many instances in which new structures are formed for the maintenance of the body, where there is no model according to which the new matter may shape itself, nor any parent-cell from which a re-producing germ may spring. Such are all the instances of formation for which, in a former course of lectures, I suggested the term of "nutritive repetition," to distinguish them from the examples of nutritive reproduction such as I have just mentioned. I then took, as an example, the different modes in which sets of teeth may succeed each other, the human second tooth being formed by materials given off from the capsule of the first; but in such jaws as the shark's, in which we see row after row of teeth succeeding each other, the row behind is not formed of germs derived from the row before, but the front row is repeated in the second, the second in the third, and so on. And again, in another class of cases the new blood-corpuscles, that are being constantly formed for the renovation of the blood, are not developed from germs given off from the old ones; neither are they formed by any assimilative force exercised by the old ones. The development of each is an independent repetition of the process by which the first was formed. And so with the successive developments of ova and epithelial cells, and many others,—each is developed independent of the rest, and each repeats the changes through which its predecessor passed. These are only examples of a large class of cases; and, numerous as they are, the theory of nutrition by assimilation, or by the development of successive cytoblasts, is inapplicable to them all. But even in instances in which we are quite sure of the operation of an assimilative force, or the existence of cytoblasts or tissue germs, we must also admit that these are controlled by the same power derived from the embryo germ. I will adduce but two examples. I have mentioned the persistence of alterations once produced by disease in the blood or any tissue; but we know that after a time these alterations are often obliterated, and the blood may so return to its perfect state as to be again amenable to the influence of poison. By what power? Certainly not by that of assimilation. It must produce the same image. By what power, then, is the natural state restored? Surely it must be by the operation of the germ power, which was for a time subjugated, but which now again brings the diseased part to the same condition as it would have been in, had no morbid change been effected in it. Again, we speak of the germs of tissues as enough to explain the formation or reproduction of the second tooth, and the successive generations of hairs, of muscular fibres, and some other tissues. But, to take the second tooth for an example of this class of cases; it is not like the first, from whose formative organs it derived its germ, but like the corresponding second tooth of the parent. What, then, determines the difference? Not, surely, the independent power of the germ or gemmule derived from the sac of the first tooth, for it is the essential character of germs to imitate those forms from which they sprang. On the whole, then, since we see that the continued mutation of particles for the growth and maintenance of the living body by nutrition manifests, in all essential things, the peculiar features that characterized the first formation of the same body from the germ, we seem justified in holding that it is one and the same power which, being maintained continuously from the germ to the latest period of normal life, determines all organic formation.

If what has been said appear sufficient to prove the operation of the germ-power in the ordinary maintenance of the body, absorbed or cast off at the term of their natural life, then more than sufficient evidence for the same opinion will appear in the phenomena of repair and reproduction after injuries. These, occupying, as it were, a middle place between the phenomena of embryonic development and those of ordinary nutrition, have of late been alternately, or in different instances, referred to one or the other of the different powers by which these phenomena are supposed to be governed. But, probably, the likeness of the phenomena of reproduction to those of both nutrition and embryonic development should have suggested that the three are accompanied by manifestations of one and the same power. That they are so, will be evident, I think, in what I shall have to describe in the following lectures, namely, that repair and reproduction cannot be in any case explained as the result of assimilation or by the succession of tissue-germs. And, secondly, if we watch the manner in which the recovery is achieved, we shall discern in it all those characters which mark the operations of the power of the germ; that in this re-formation there is an independent, though concurrent, development of many parts, and that the whole process is limited by the same restrictions of time, and space, and exact method, as those observed in the original development of the parts.—*Medical Times.*

[To be continued.]

